# Influence of Oyster Mushroom Waste on Growth Performance, Immunity and Intestinal Morphology Compared With Antibiotics in Broiler Chickens

**DOI:** 10.3389/fvets.2020.00333

**Published:** 2020-06-11

**Authors:** Reda A. Hassan, Manal E. Shafi, Khalil M. Attia, Mohamed H. Assar

**Affiliations:** ^1^Animal Production Research Institute, Agricultural Research Center, Ministry of Agriculture, Giza, Egypt; ^2^Department of Biological Sciences, Zoology, King Abdulaziz University, Jeddah, Saudi Arabia

**Keywords:** oyster mushroom waste, broiler chickens, responses, glutathione, glutathione peroxidase

## Abstract

Oyster mushroom waste (OMW) is a by-product of the agriculture industry with valuable antimicrobial, antioxidant, antifungal, and prebiotic properties. This by-product might be a useful alternative to antibiotic growth stimulators in poultry nutrition. The purpose of this research was to test the impact of OMW on the immune responses and on the morphology of intestine of broiler chickens. Four dietary therapies with five replicas of 15 birds in each, totalling 300 day- Ross 308 broiler chickens, were utilized in this study. Control chickens were fed a mixed diet that included a maize-soybean meal complemented by 1 and 2% OMW in addition to the basal diet. Furthermore, Enramycin (125 g/kg) was added to the control diet as an antibiotic. Throughout this experiment, performance was studied as well as the immune response to the Newcastle Disease Virus (NDV) and intestinal morphological traits. A substantial surge was noted in body weight gain (BWG) and feed intake (FI) of chickens after the addition of 1% OMW (*p* ≤ 0.05). In contrast, feed supplementation with 2% OMW, compared with the control diet, produced no noteworthy increase in BWG or the feed conversion rate (FCR). Antibiotic addition, on the other hand, increased serum cholesterol (*p* ≤ 0.05). After 42 days, neither OMW nor antibiotic addition affected organ mass. In contrast, antibiotic addition reduced the small intestine percentage, crypt depth and villus height (*p* ≤ 0.05). The Newcastle disease vaccine (NDV) antibody titer improved after feed supplementation with 1% OMW comparing with the control and antibiotic diet group. Furthermore, OMW supplementation decreased the heterophil-to-lymphocyte H/L ratio (*p* ≤ 0.05). The use of OMW led to a reduction in the malondialdehyde (MDA) content of the breast and liver and an increase in glutathione peroxidase. It helped to reduce glutathione, glutathione reductase, and glutathione S-transferase. In conclusion, the impact of OMW were dose-dependent, and the use of 1% OMW in broiler diets enhanced their growth and immunity. Nonetheless, supplementation with 2% OMW produced conflicting results.

## Introduction

Antibiotics have historically been used as promotors of growth in poultry feed and as treatment for contaminated chickens. For decades, conventional artificial nutritional additives, like antibiotic growth promoters, antioxidants, antifungal agents, and anti-parasitic agents are used in poultry feed (1). However, long-term, and unnecessary use of antibiotics in the poultry diet will result in hazards effects on animal and human health. Zhang et al. (2) and Roy and Fahim (3) stated that, in agricultural processing, antibiotic-resistant bacteria are produced when overusing antibiotics in food as well as in food products derived from animals, thereby exposing humans to these bacteria. Antibiotics have been limited in many countries to be used as growth promoters in animal feed and poultry diets (4, 5). Currently Poultry researchers are using experimental additives of natural basis to substitute antibiotics. This substitution has proved to be an effective therapy to boost health status and efficiency in development. Now animal researchers are looking for possible natural feed options that may increase livestock and poultry production efficiency and health status (5, 6). Mushroom waste in the poultry industry could be a suitable replacement for antibiotics (6). It is available from mushroom farms, and possesses prebiotic, antimicrobial, antifungal, and antioxidant properties (7).

Worldwide, feed supplies for animal nutrition are in shortage, the problem get even difficult after COVID-19 due to many consequence negative effects on crop cultivation, harvest and production as well as trade. Hence, the use of agro-industry as feedstuffs in animal nutrition represents an important source of protein, vitamins, minerals and antioxidants and visible solution (3–5). This process can decrease the environmental pollution and enhance the efficiency of production, farm profit and quality of animal product due to bioactive substance in OMW. In addition, the alternative natural pharmaceutical products that have originated from plants have been used as feed supplements for centuries in veterinary medicine (8). The use of OMW as feed stuffs in the animal feeding may also be enhance the performance due to several bioactive substances (2–6).

*Pleurotus ostreatus* stem waste is abundantly available due to rise in the mushroom's cultivation. The stems are treated as agricultural by-product, although they have medicinal and nutritive values. The popularity of mushrooms for human consumption has also lead to environmental pollution (6–9). However, to date, the use of *Pleurotus ostreatus* stem waste in the farm animals' production has been minimal. An *in vitro* analysis of the effects of mushroom and herbal polysaccharides on immune function may provide a theoretical basis for the potential use of these components to boost farm animal growth, health status, and economic efficiency (10). The weight of internal organs in birds is an indication of healthy digestion and good health. The weight of lymphoid organs such as spleen, liver, thymus, bursa, and lymphoid tissues could assess immune responses in avian organisms (11). Gastrointestinal tract production has a good part to play in the bird's immune system. Taking these facts into consideration, this study examined the suitability of mushroom (*Pleurotus ostreatus*) stem waste as a phytogenic feed additive and substitute for antibiotics in broilers diets. In general, there have been insufficient studies in the examination of the health status and performance in broilers due to the efficiency of mushroom waste. For long term, antibiotics have been overused in the industry of poultry to raise production efficiency and health status has resulted in hazards effects on human health. This work investigates medicinal mushrooms usage in poultry feed dispose of antibiotics, without impacting optimal efficiency. Biologically active substances are found in many medicinal mushrooms like glycoproteins, polysaccharides, and other polymers that may function as preferable nutritional supplements in chickens as well as immunomodulating agents. The aim of the research was, therefore, to test the effectiveness of *Pleurotus ostreatus* oyster mushroom waste (OMW) in broiler chickens on growth output, serum cholesterol, immune status, and antibody response.

## Materials and Methods

The experimental protocol of the present work was approved by King Abdulaziz University, Jeddah, Saudi Arabia. The University recommend that animal protections, welfare, rights, and minimum stress levels, and avoided any suffering or harm to creatures according to Royal Decree number M59 in 14/9/1431H.

### Management and Birds

An overall number of 300 unsexed 1-day old chicks were assigned to be fed four different experimental treatments randomly (Ross 308). Five duplicated pens with 15 birds in each pen with a ratio of 1:1 for each treatment were chosen. All birds in this study were raised on a commercial poultry farm and kept in-floor pens that have litters made of wood shreds. A totally randomized design was used in this experiment, following are the dietary treatments: (1) as control, a diet consisting of maize-soybean-based was used as a meal, (2) 1% OMW control diet supplementation, (3) 2% OMW supplemented to control diet and (4) control diet with Enramycin (125 g/kg) as a growth promoter effective against gram-positive bacteria. The control nutrition schedule was designed to meet or surpass the mandated nutrient requirements (12). The chickens' feeding schedule included fed starter, and finisher diets served in the form of mash. This feeding process was followed through day 0–21 as well as day 22–42, respectively. During the entire trial, ad libitum feed and water were given. On day 21, gradually the pen's temperature had been reduced from 33 to 25°C, then adjusted and stabilized until the end of the experiment. All experimental food ingredients and nutrient requirements used in this trial are shown in Table 1.

**Table 1:** The profile, calculated and determined analysis of the starting and finishing diets.

**Ingredients, %**	**Starter diets**			**Finisher diets**		
	**Control**	**1% OMW**	**2% OMW**	**Control**	**1% OMW**	**2% OMW**
Yellow corn	53.7	53.2	52.4	62.2	61.5	61.0
Soybean meal (44%)	26.0	26.5	26.3	22.3	22.0	21.2
Wheat bran	4.0	3.0	3.0	0.0	0.0	0.0
Corn gluten meal	10.2	10.2	10.2	8.8	8.8	9.1
Vegetable oil	2.1	2.1	2.1	3.0	3.0	3.0
Mushroom waste	0.0	1.0	2.0	0.0	1.0	2.0
Di-calcium phosphate	1.8	1.8	1.8	1.5	1.5	1.5
Limestone	1.3	1.3	1.3	1.4	1.4	1.4
Dl-methionine	0.2	0.2	0.2	0.1	0.1	0.1
L-lysine	0.1	0.1	0.1	0.1	0.1	0.1
NaCl	0.3	0.3	0.3	0.3	0.3	0.3
Premix^*^	0.3	0.3	0.3	0.3	0.3	0.3
Total	100	100	100	100	100	100
CALCULATED VALUES:^**^						
Metabolizable energy (Kcal/kg)	3000	3000	3000	3178	3173	3175
Calcium (%)	1.0	1.0	1.0	0.93	0.93	0.93
Available phosphorus (%)	0.49	0.49	0.49	0.42	0.42	0.42
Lysine (%)	1.12	1.12	1.12	0.99	0.98	0.96
Methionine (%)	0.68	0.68	0.68	0.54	0.53	0.53
**CHEMICAL ANALYSIS (%)**						
Dry matter	85.8	85.68	85.58	85.38	85.42	85.60
Crude protein (CP%)	22.92	22.90	22.96	20.5	20.7	20.3
Ether extract (EE%)	2.62	2.58	2.55	2.66	2.63	2.60
Crude fibre (CF%)	3.68	3.55	3.50	3.15	3.09	3.03
Ash	6.78	6.72	6.70	6.55	6.60	6.52

* *vit. & Min. mix: each 3 kg contains: vit. A, 12,000,000 IU; vit. D3, 2,000,000 IU; vit. E, 10 g; vit. K, 2.0 g; vit. B1, 1 g; vit. B2, 5 g; vit. B6, 1.5 g; vit. B12, 10 mg; folic acid, 1 g; biotin, 50 mg; pantothenic acid, 10 g; nicotinic acid, 30g; choline chloride, 250 g; Mn, 60 g; Fe, 30 g; Zn, 50 g; Cu, 10 g; I, 1 g; Co, 100 mg; Se, 100 mg and complete to 3.0 kg by calcium carbonate*.

** *Calculated according to National research council (12)*.

### Mushroom Waste Preparation

Mushroom waste consisted mainly of oyster mushroom stem base (*Pleurotus osteratus*) and mushroom growth plan rejections. Mushroom waste was prepared from the base of the stem of the mushroom. After harvesting, the mushrooms were weighed, placed in net bags, air/sun-dried, and eventually, the OMW was collected. After the sun drying, when a substantial volume of water was lost, OMW was further exposed to oven drying to eliminate water thoroughly. Upon drying in the oven, mushrooms were held at a consistent temperature of 60°C, and once the samples showed a steady weight, the process was stopped.

Using a feed grinder, oven-dried OMW was smashed to a small size resembling the commercial feed particles with which it was eventually mixed. At this stage, the OMW resulting from the earlier process was then stored in a sealed and transparent storage container before being mixed into the basal feeds.

### Chemical Analysis

All the resulting OMWs were first frozen and dried via a 1-mm sieve, after that analyses of fiber, fat, ash and, protein were performed as directed (13). Overall, the content of protein was determined using the Kjeldahl method (13). From petroleum ether samples, crude fat content was extracted in a Soxhlet apparatus. While the content of crude fiber in a Dosi Fiber apparatus was investigated (13) Through incineration in a furnace for 6 h at 600°C and by drying in an oven, dried samples were turned into ash. Following an aqua regia digestion phase, the calcium and phosphorus content of OMW was analyzed and examined by inductively coupled plasma (ICP) and optical emission spectroscopy. Table 2 exhibits the results of these analyses.

**Table 2:** Chemical composition of the oyster mushroom waste used in the current research (on percent air dried weight).

**CP, %**	**CF, %**	**EE, %**	**Ash, %**	**Moisture, %**	**Ca, %**	**P, %**	**NFE, %**	**ME, kcal/kg**
8.9	33.1	0.46	3.7	5.3	0.08	0.30	48.54	2520^*^

* *Calculated according to Janssen (14)*.

### Performance Parameters

In this experiment both body weight and food intake were controlled frequently over a pen-based foundation. However, body mass increase and feed conversion rate values were calculated afterward. The death rate in each pen was reported daily.

### Blood Parameters

At the age of 42 days, three birds were bled from each treatment. To obtain serum, for ~15 min blood samples were centrifuged at 3,500 rpm. The samples were then frozen for further analysis at −20°C. Serum was extracted to determine total cholesterol (TC), total triglycerides (TG), and high- and very-low-density lipoprotein cholesterol (HDL-C and VLDL-C). A spectrophotometer was used to measure serum lipids. Biochemical analysis of serum samples was performed using commercial diagnostic kits (Egyptian Biotechnology Company, Cairo, Egypt), and VLDL-C determination was carried out by dividing TC by 5.

The H/L ratio was calculated from a blood sampling obtained on day 42. In this procedure, heparin-containing syringes were used to prevent the development of blood clots. Greenwald, May, and Giemsa dyes were used to stain blood smears (15). Under optical microscope, one hundred leukocytes for every specimen were calculated and documented (16) by heterophil-to-lymphocyte separation and lymphocyte heterophil (H/L) percentage. Using ELISA test kits, levels of immunoglobulin M (IgM), immunoglobulin, A (IgA) serum, immunoglobulin G (IgG) serum were measured at a wavelength of 450 nm (Shanghai Lengton Biosciences Co. Ltd., Shanghai, China).

Birds used in this experiment were vaccinated individually using an eyedropper with Newcastle Disease Virus (NDV) commercial vaccine at 6-day age, and in drinking water at the ages of 16 and 26 days. Also, three chickens from each treatment were bled at the age of 42 days to obtain serum for NDV antibody measurement. As defined (17), antibody titers were evaluated using the hemagglutination inhibition (HI) test against NDV, while the HI titer was viewed as the highest reciprocal value of the dilution.

### Lymphoid Organ Relative Weight and Small Intestinal Morphology

Three chickens at age of 42 days, were selected from every pen, then were slaughtered according to the Islamic (Halal) method (18). Organs (livers, spleen, and bursa), small intestine parts (ileum, jejunum and duodenum), and abdominal fat were detached, cleaned, measured, then reported as a proportion of live bird's body weight at slaughter. The estimated length in parts of the small intestine was stated as a percentage of overall intestinal length. Samples were taken directly from the jejunum in small intestine, halfway between the point of entry of the Meckel's diverticulum and the bile ducts. From the ileocecal junction, 10 cm of ileum were represented, which was obtained and to measure the crypt depth, crypt width, and villus height. The 1.5 cm long segments were washed using saline solution before fixing in 100 g l-1 formalin buffer (pH 7.0). For microscopic testing, the paraffin-coated samples of intestine were then sectioned to a 5 μm thickness before being stained by hematoxylin-eosin.

The measurement of villus height (micrometers) started from villus's edge until the villus-crypt junction, while depth of the crypt was measured starting at the base until the transition area amongst the crypt and villus. Tissues of the liver, breast, and thigh muscle were collected after slaughter by eliminating the connective tissue, skin, and fat. Specimens were packed in vacuum at −40°C to prepare them for processing. With regarding to measuring the antioxidant, tissues were defrosted than processed for five days at 4°C. Afterward, they were subjected to antioxidant activity and the oxidation of lipid in storage at 0 and 5 d. In this experiment, many antioxidants were measured (reduced glutathione (GSH), glutathione reductase (GR), glutathione peroxidase (GSH-Px), glutathione S-transferase (GST), and malondialdehyde (MDA).

Using the approaches of (19), glutathione peroxidase's activity was tested. Reduced glutathione was calculated by adapting the procedure in Paglia and Valentine (20). The activity of glutathione reductase was calculated according to Staal et al. (21). Using the method outlined in Habig et al. (22), the activity of glutathione S-transferase was measured. The technique described by Buege and Aust (23) used malondialdehyde as a peroxidation marker. The method of Bradford (24) was used to determine tissue proteins.

### Statistical Analysis

One Way 2008 SAS statistical model was utilized in this study to analyse the variance in data appropriate for a completely randomized model were analyzed according to SAS Institute (25). Using the multiple-range test, small differences between treatment methods were tested (26), and significant differences were considered at *p* ≤ 0.05.

Model:

$$\begin{array}{l} X_{ij}=u+\;T_{i\;}+e_{ij} \end{array}$$

Where:

$$\begin{array}{l} X_{ij}=Any\;observation\;\\ u=Overall\;mean\\ T_{i\;}=Treatments\left(i=1,2\ldots and\;4\right)\\ e_{ij}=Experimental\;error \end{array}$$

## Results and Discussion

### Performance Parameters

A summary of the effect of experimental treatments in birds on BWG, FI, and FCR is shown in Table 3. Supplementation with 1% OMW and antibiotic increased BWG and improved FCR in broiler chickens relative to the un-treated group (*p* ≤ 0.05). However, supplementing broiler with 1% OMW resulted in greater BWG than the control and other treatments. In contrast, BW was not different when fed 2% OMW compared with the control. Supplementation with 1% OMW resulted in a noteworthy surge in FI (*p* ≤ 0.05) during overall times, whereas no effect of 2% OMW was observed. There was no effect of Enramycin on FI relative to the control (*p* ≤ 0.05). The improvement in FI seen in chickens fed the diet supplemented with 1% OMW could be the explanation for the significant increase observed in broiler BW. However, this increase may also be attributed to the presence of oligosaccharides in the cell walls of mushrooms (3–7). They could have influenced the equilibrium of the intestinal tract microflora positively by functioning as growth stimulators and prebiotics (27). Feed efficiency and body weight (Table 3; compared with the control group) were effected favorably using OMW, this may be due to the inclusion of mushroom positively affecting balance of microflora in chicken's intestinal tract leading to a more effective utilization of dietary nutrients. Feeds supplemented with mushroom and herbal extract decreased Bacteroides spp., *E. Coli*, and Enterococci numbers while the numbers of Lactobacilli and Bifidobacteria in broilers gut; our inferences could be further confirmed (28). However, large intestine contains kinds of bacteria which are active in the hydrolysis of macro-molecules carbohydrates such as oligo- and polysaccharides, creating micro-molecular weight in carbohydrates from macro-polymers before fermenting them, leading to higher count of bacteria (3–7). The residual product of fermentation, such as lower intestinal pH and short-chain fatty acids, can inhibit hazardous bacteria and cause stimulation to beneficial bacteria (29). This effect is especially needed at the sensitive stage when chicks are more vulnerable and exposed to intestinal pathogens. This shows that the older birds may have decreased nutritional with age, and they have a more evolved digestion in tract and organs (3–5).

**Table 3:** Effect of dietary treatments on body weight, ADG, ADFI and the FCR of broiler chicks.

**Parameters**	**Dietary treatments**				**SEM**	***P* values**
	**Control**	**1% OMW**	**2% OMW**	**Antibiotic**		
**BODY WEIGHT, G**						
21 days	657^b^	752^a^	655^b^	735^ab^	8.9	0.014
42 days	2503^b^	2685^a^	2500^b^	2648^ab^	21.3	0.035
**STARTER PERIOD, THE AGE OF 1 TO 21 DAYS**						
ADG, g	31.3^b^	35.8^a^	31.2^b^	35.0^ab^	0.12	0.045
ADFI, g	49.8	52.1	50.0	51.3	0.35	0.118
FCR, g/g	1.59^a^	1.45^b^	1.60^a^	1.47^b^	0.014	0.006
**FINISHER PERIOD, THE AGE OF 22 TO 42 DAYS**						
ADG, g	87.9^b^	92.1^a^	87.9^b^	91.1^ab^	0.22	0.037
ADFI, g	160.4^b^	163.1^a^	160.0^b^	162.5^a^	1.26	0.028
FCR, g/g	1.82^a^	1.77^b^	1.82^a^	1.78^b^	0.05	0.005
**OVERALL, THE AGE OF 1 TO 42 DAYS**						
ADG, g	59.6^b^	63.9^a^	59.5^b^	63.1^a^	0.41	0.029
ADFI, g	105.1^b^	107.6^a^	105.0^b^	106.9^a^	0.52	0.035
FCR, g/g	1.76^a^	1.68^b^	1.76^a^	1.70^b^	0.02	0.002
**DIGESTIBILITY**						
CP, %	54.6^b^	59.0^a^	54.5^b^	56.8^ab^	4.456	0.042
OM, %	71.8	75.5	72.0	73.60	2.722	0.114

a,b *Means for each trait at p < 0.05 significant difference in the different superscripts; OMW, mushroom waste; ADG, average daily gain; ADFI, average daily feed intake; FCR, feed conversion rate; CP, crude protein; OM, organic matter*.

Chicken BW was not affected by feed supplementation with 2% OMW throughout the entire experiment. The reason resulting in the fiber-rich content of OMW led to a reduction of nutrient utilization in chickens. Unlike this study, Willis et al. (30) demonstrated that chickens displayed a marked surge in BW using 5% oyster mushroom feed as a supplement at day 49 of age. In contrast, Toghyani et al. (31) reported that 2% of mushroom feed resulted in decreased chicken BW compared with 1% addition under the current study.

Adding 1% OMW and Enramycin enhanced (*p* ≤ 0.05) FCR comparing with control conditions. However, for the entire experimental period, 2% OMW did not affect this ratio (*p* ≤ 0.05). In comparison with the control, Enramycin was found to considerably increased LBW, ADFI, and ADG, and improved FCR. Antibiotics effectively boost growth efficiency by altering the gastrointestinal microbiota (in particular by destroying pathogens), thus reducing inflammation of the intestinal mucosa (32), which has a significant effect on nutrient absorption.

The use of 2% OMW in combination with antibiotic treatment was not found effective against the retention of organic matter and protein relative to regulation, whereas 1% OMW increased the digestion of crude protein and organic matter (Table 3). The higher CP digestibility in broilers with OMW might be associated with significantly higher final live weight in OMW groups in this study. A previous study (16, 18, 33–35) did not show any beneficial effect of antibiotics on the nutrient used in broiler chickens, in accordance with this observation. On the other hand, a mixture of oyster mushroom, propolis, and garlic extract did not impair nutrient digestibility compared with antibiotics (16). The impact of feed additives and/or phytogenic plants may vary among different experiments due to strain and age of chickens, environmental and housing conditions, type and concentrations of feed additives, and hygienic conditions (18, 33, 34).

### Blood Parameters

The data in Table 4 show the effect of OMW and Enramycin on the serum metabolic profile. Antibiotics increased TC relative to the control, whereas the addition of 1 or 2% OMW decreased TC after the trial (Table 4). In contrast to the un-treatment group, by the end of this research, experimental additives did not affect TG, HDL-C, or VLDL-C. This outcome is in agreement with observations in the literature that the dietary use of mushrooms reduces serum cholesterol (36). Moreover, another study (37) found that at both levels of mushroom (*Flammulina velutipes*) stem waste supplementation (1 and 2%), TC decreased relative to the control. At both levels of OMW supplementation, TC was recorded to be (p < 0.05) which is lower than antibiotic-supplemented groups and the control groups. In this study, no substantial change was witnessed in low-density lipoprotein cholesterol (LDL-C) nor TG.

**Table 4:** Effect of dietary treatments on some parameters of blood and the immune response in 42- day-old of broiler chicks.

**Parameters**	**Dietary treatments**				**SEM**	***P* value**
	**Control**	**1% OMW**	**2% OMW**	**Antibiotic**		
**PARAMETERS, MG/DL**						
TG	96.35	93.00	93.60	94.85	0.112	0.458
CHL	95.25^b^	80.60^bc^	75.50^c^	120.10^a^	0.225	0.002
HDL-C	45.85	46.20	46.36	50.00	0.525	0.446
VLDL-C	19.27	17.60	17.72	18.97	0.125	0.457
TP, g/dl	2.62	2.74	2.70	2.68	0.116	0.605
ALB, g/dl	1.63	1.72	1.70	1.69	0.076	0.615
BUN	3.21	3.18	3.20	3.26	0.42	0.610
**IMMUNE RESPONSE**						
NDV, log2	6.41^b^	6.94^a^	6.55^b^	6.54^b^	0.452	0.047
IgG	1.50^b^	2.27^a^	1.48^b^	2.05^a^	0.123	0.025
IgM	1.41^b^	2.86^a^	1.50^b^	2.53^a^	0.152	0.005
H/L	0.617^a^	0.587^b^	0.476^c^	0.589^b^	0.058	0.0001

a,b,c *Means for each trait with different superscripts differ significantly at p < 0.05; OMW, mushroom waste; CHL, cholesterol; TG, triglycerides; HDL-C, high-density lipoprotein cholesterol; VLDL-C, very-low density lipoprotein cholesterol; TP, total protein, ALB, albumin; BUN, blood urea nitrogen, NDV, Newcastle disease virus H/L heterophil-to-lymphocyte ratio*.

Moreover, no statistically significant differences were found for blood total protein, total albumin, globulin, or urea nitrogen concentrations at day 42 between treatment groups and control (Table 4). Ultimately, therapies have failed to cause any major effect on the concentrations of serum of total globulin, or albumin, and protein. In this analysis, it can explain why there was no apparent effect of mushrooms on these compounds in the blood. Similarly, Guo et al. (10) indicated that under infectious conditions, the effect on mushrooms was more pronounced than under normal ones.

In the present study, OMW was used as a dietary supplement effectively decreased TC. Edible mushrooms have been proposed as an oral medicine due to their hypocholesterolemic effect (38). High serum TG and LDL-C rates have been documented in connection with an increased hazard of disorders like increased abdominal adiposity and fatty liver in metabolic system in birds (38). Our results in this study were comparable with those of Shang et al. (39), who stated that all levels of TG, TC, and LDL-C were lower in broilers fed a mushroom diet compared to the control diet. Furthermore, adding mushroom (*Agaricus blazei*) powder has been shown to reduce TC, yet it had no influence on serum TG in broilers (40). Moreover, broilers fed diets supplemented with mushroom (*Pleurotus ostreatus*), TC was reduced, yet other serum lipids were not affected (41). In contrast, Simon et al. (42) indicated that in OMW-fed broilers, serum TG was lower than in controls. Nevertheless, in their experiment, TG, HDL-C, and VLDL-C, did not vary between the treatments. The high quantity of natural fiber in OMW could have a role in reducing TC via an increase in lipid metabolism in chickens.

In literature a positive effect of mushroom on lipid metabolism in male hamsters was reported by Yeh et al. (43). Their research found, the extract and the powder from needle mushrooms could reduce cholesterol levels in hamsters in the serum and liver tissue. In another research by (44) observed a lower total cholesterol (TC), low-density lipoprotein cholesterol and plasma triglyceride in diet-induced hyperlipidemic rats being fed Hericiumerinaceus mushroom exo-polymer. Lovastatin and λ-aminobutyric acid (GABA) have been reported from fruiting bodies of *F. velutipes* (45). Lovastatin is used to minimize the development of cholesterol, which may minimize the risk of heart disease (45, 46). Another research by Harada et al. (47) published very successful results by using GABA-mediated *F velutipes* Mushroom powder to decrease the systolic pressure in rats. β-D-glucan and its by-products in medicinal mushrooms result in decreasing the impacts of cholesterol via decreasing absorption or through fecal elimination (9). Also popular for its cholesterol-reducing functions is the oyster mushroom (48).

Table 4 shows the influence of mushroom stem by-product on serum metabolic profile, antibody titers alongside NDV; the status of immune serum and the H/L ratio are shown as well.

Antibody titers comparing to ND virus vaccines were higher (*P* < 0.05) in OMW diets comparing to those fed with the antibiotic and control diets. The optimal responses for antibody titer were found in broiler fed 1% OMW (Table 4). Broilers illustrated higher (*P* < 0.05) in OMW and antibiotic groups illustrated greater (*p* < 0.05) serum immunoglobulin (IgG and IgM) levels than the control and group (Table 4). The highest concentrations for immunoglobulin parameters were observed in the 1% OMW group. The concentration of Serum immunoglobulin may create a humoral immune response in animals owing to their vital characters in the immune roles in combating against infection (49). Antibody titers for avian influenza (AI), infectious bronchitis (IB) and Newcastle disease (ND) vaccines for the virus were noted to be higher (*P* < 0,05) in pullet-fed diets of oyster mushroom waste (6–16, 16–18, 18–37). Furthermore, in mushroom-fed diets, the parameters (IgG, IgM, and IgA) of serum immunoglobulin were observed to be greater (*p* < 0.05) comparing to the antibiotic-fed and control diets in the pullet of experiment. Bai et al. (49) reported that concentrations of serum immunoglobulin could produce a humoral immune response in samples because of their essential role in the battle against various infections through immune function. Additionally, β-glucan supplementation from consumable mushroom has had a major immune-stimulating impact in boilers (50). Likewise, in mushroom Ganoderma lucidum fed populations, the antibody titers opposing to infectious bursal disease virus were more significant than the control diets in pullet (51). This analysis further showed that the concentrations of cytokines (IL-4, IL-2, IL-6, and TNF-α) of serum in mushroom feed groups were higher (p < 0.05) than those of un-treated and antibiotic feed groups in laying hens. In laboratory samples, it was found that Mushroom polysaccharides generate various cytokines and raise the immune-stimulating organ's weight (52, 53). The polysaccharides in mushroom increased body weight and thymus and spleen's weight ratio in experimental mice as well as it may have modulated the thymocyte and splenocyte T cell subpopulation (31). Moreover, mushroom polysaccharides increased the development of TNF-a, IL-6 and IL-1b, NO (nitric oxide), and mice model lymphocyte proliferation (54).

Mushrooms contain compounds that stimulate the immune system, for example, glycosides, alkaloids, polysaccharides, organic acids, and volatile oils (55, 56). Research studies illustrated that both adaptive and innate immunity including humoral and cellular responses could be affected by the present of poly and oligosaccharides in mushroom (57). Furthermore, mushrooms are rich in selenium, a trace element that increases the antioxidant potential (58) and consequently improves the immunity of animals that consume it. Furthermore, polysaccharides extracted from mushrooms have displayed protective effects against the infection of *E. tenella* (59). Interestingly, even though mushrooms have been shown to exert beneficial effects on the immune system, chickens fed a diet containing 2% mushroom extract in this trial displayed no impact on the antibody titer against NDV in comparison with the control group. Even though humoral immunity working against NDV was expected to increase, further studies on this topic are needed (39). The addition of 3% mushroom extract has also been proposed to enhance antibody titers against SRBC (58, 59). The positive influence on the immune response from antibiotics has additionally been documented. Another study (32) found that Flavomycin may induce a high-level response of the antibody to NDV, yet it did not affect the development of immunoglobulins relative to the birds in the control group. According to (7), supplementation with 2% mushroom extract could reduce the antibody titer against ND but, could increase antibody titers against the Avian Influenza (AI) virus. The latter result suggests a reinvestigation of antibody titers with the inclusion of OMW in broilers' diets.

Table 4 shows that the H/L ratio declined at both levels of OMW supplementation, especially the 2% level (*p* ≤ 0.05). The physiological stress indicator is the ratio of heterophils to lymphocytes as the number of heterophils increases in moderately stressful settings (60). Since there was no risk of disease in this study, the H/L ratio was not expected to increase. Consequently, a decrease in this ratio could indicate that no stress was imposed on the OMW-treated group. Vetter and Lelley (58) presented a marginal decline in this ratio, and this in agreement with our results.

### Intestinal Morphology

Table 5 summarizes the intestinal morphology of controls and supplemented poultry. The relative length of small intestine fragments was not influenced by OMW and antibiotics when comparing with the control diet in 42-day-old broilers (Table 5). Birds with antibiotics included in their diets had lower relative ileum, jejunum and duodenum weights (*p* ≤ 0.05) relative to control birds at 42 days of age, even though OMWs did not affect (*p* ≤ 0.05).

**Table 5:** Impact of various levels of oyster mushroom waste on lymphoid organ relative weight and intestinal morphology of broiler chickens at the age of 42 days.

**Parameters**	**Dietary treatments**				**SEM**	***P* value**
	**Control**	**1% OMW**	**2% OMW**	**Antibiotic**		
**RELATIVE LENGTHS, % OF SMALL INTESTINE LENGTH**						
Duodenum	17.25	17.88	17.80	17.30	0.143	0.931
Jejunum	39.00	39.36	39.50	41.00	2.873	0.103
Ileum	43.00	42.70	41.63	41.00	0.693	0.582
**RELATIVE WEIGHT, % OF LIVE BODY WEIGHT**						
Duodenum	0.88^a^	0.78^a^	0.81^a^	0.60^b^	15.661	0.001
Jejunum	1.38^a^	1.40^a^	1.46^a^	0.98^b^	3.55	0.046
Ileum	1.13^a^	1.16^a^	1.13^a^	0.90^b^	3.859	0.002
**JEJUNUM**						
Villus height, μm	1050^b^	1240^a^	1220^a^	1100^b^	7.028	0.012
Crypt depth, μm	185^b^	188^ab^	210^a^	183^b^	5.609	0.023
V/C	5.68	6.26	5.82	6.01	3.142	0.87
**ILEUM**						
Villus height, μm	685^a^	510^b^	518^b^	680^a^	108.43	0.0001
Crypt depth, μm	190^a^	160^b^	163^b^	195^a^	15.65	0.001
V/C	3.61^a^	3.19^b^	3.19^b^	3.49^ab^	4.067	0.050
**LYMPHOID ORGANS, %**						
Spleen	0.132	0.130	0.132	0.133	0.950	0.461
Bursa	0.145	0.148	0.142	0.146	2.716	0.115
**CARCASS TRAITS, % BW**						
Carcass	62.90	63.96	63.00	63.85	2.879	0.103
Liver	2.47	2.40	2.45	2.59	1.459	0.297
Abdominal fat	1.05	1.08	1.05	1.03	0.467	0.713

a,b,c *Means for each trait with different superscripts differ significantly at p < 0.05; OMW, mushroom waste*.

A considerable increase of villus height and crypt depth of the jejunum between 1 and 2% OMW inclusion was noticed. In contrast, all levels of OMW supplementation (*p* ≤ 0.05) decline these indices in the ileum. Given the variations in villus height and crypt depth of the jejunum, no significant effect of OMW was observed on the ratio of villus height to crypt depth, although this ratio was amplified in the ileum (*p* ≤ 0.05). The safety and health of the gut and improved absorption ability of the intestines were attributed and correlated to the higher villus height and lower crypt depth. In this analysis, the high fiber content of mushroom may have led to structural and body alternations in the small intestines of birds, but no impact the ratio of villus height to crypt depth was noticed. Gross and Siegel (61) reported that birds fed 1 and 2% OMW had an increase in bird villus height in the duodenum, ileum, and jejunum; this result is relative to the results obtained from the current jejunum study.

Relative weights of the jejunum, ileum and duodenum were lowered by antibiotics, whereas mushrooms did not change the weight and length of small intestine segments. Adibmoradi et al. (62) confirmed the ability of the microbial population in the gut of broilers to rise the production of volatile fatty acids (VFAs) and polyamines that attach both to the sites of absorption in small intestine. VFAs enhance gastrointestinal wall thickness and thus contribute to raising the weight of the small intestine.

Stopping the population growth of microbial gut, as seen in recent study, helps antibiotics reducing the microbial development of polyamines and VFAs, consequently leading to a decrease in the bowel weight. In the current trial, the inclusion of OMW, particularly 1%, in broiler diets likely improved intestinal nutrient absorption, possibly due to increased villus height in the intestine.

### Relative Lymphoid Organ Weight

Table 5 similarly shows the effects on lymphoid organs of the control group and mushroom or antibiotic supplementation. At 42 days of age, OMW or antibiotics did not compromise any of the lymphoid organs. Organ relative weight was neither affected by mushroom nor antibiotic. In other relative organs the weights were not affected by OMW inclusion, which ensured that the average health of broilers consuming OMW was not impaired during the experimental period and that more excellent immunity was gained. In some studies, it was reported that mushroom had no significant effect on lymphoid organ weight (7–16, 16–18, 18–63).

A higher bursa weight usually represents a better anatomical response and superior health in the face of changes in immunity due to stress (30). Many authors have previously pointed out the absence of an effect of mushrooms on lymphoid organs (66–31).

### Antioxidant Parameters

Table 6 shows that chicken breast, liver, and thigh tissues had GST, GR, GSH, and GSH-Px activities that were not varied between clusters on d 0. However, during refrigerated storage step, control or antibiotic groups presented a vital reduction in GR, GST, GSH, and GSH-Px activities in parallel with the mushroom-supplemented groups on the fifth day.

**Table 6:** Effect of dietary treatments on the oxidative enzyme of the liver, thigh, and breast tissues of broiler chickens stored in refrigerator at 0 or 5 d.

**Dietary treatments**	**Liver**		**Thigh**		**Breast**	
**Storage time**	**Day 0**	**Day 5**	**Day 0**	**Day 5**	**Day 0**	**Day 5**
**GLUTATHIONE S-TRANSFERASE, MMOL/MIN/MG OF PROTEIN**						
Control	1.65	0.76^b^	0.50	0.22^b^	0.52	0.30^b^
1% OMW	2.00	1.50^a^	0.53	0.46^a^	0.70	0.65^a^
2% OMW	2.05	1.53^a^	0.49	0.36^ab^	0.68	0.60^a^
Antibiotic	1.70	0.95^ab^	0.52	0.20^b^	0.54	0.40^b^
SEM	1.504	4.749	0.040	5.947	2.60	8.188
*P* value	0.286	0.035	0.989	0.020	0.124	0.008
**REDUCED GLUTATHIONE**, **μM**						
Control	2.86	2.00^b^	2.10	1.86^b^	2.36	2.08^b^
1% OMW	4.30	4.00^a^	3.43	3.10^a^	3.86	3.70^a^
2% OMW	3.90	3.86^a^	3.00	3.00^a^	3.50	3.10^a^
Antibiotic	2.68	2.05^b^	2.20	1.70^b^	2.30	2.06^b^
SEM	1.865	3.640	1.621	5.018	2.325	2.590
*P* value	0.214	0.054	0.260	0.030	0.151	0.050
**GLUTATHIONE REDUCTASE, U/MG OF PROTEIN**						
Control	23.17	18.50^b^	14.10	10.00^b^	28.70	23.00^b^
1% OMW	26.00	23.10^a^	15.19	15.00^a^	30.50	29.20^a^
2% OMW	28.00	20.13^ab^	14.00	12.50^b^	30.80	29.17^a^
Antibiotic	25.00	20.00^ab^	13.86	11.00^b^	29.00	23.17^b^
SEM	3.043	3.424	1.115	8.107	1.025	9.927
*P* value	0.093	0.050	0.398	0.008	0.432	0.005
**GLUTATHIONE PEROXIDASE, MU/MG OF PROTEIN**						
Control	8.10	5.00^b^	10.00	6.500^b^	3.80	3.80^b^
1% OMW	10.00	9.20^a^	11.20	10.60^a^	5.20	5.16^a^
2% OMW	8.20	7.06^ab^	10.22	10.50^a^	4.86	4.62^ab^
Antibiotic	8.05	5.35^b^	10.30	6.00^b^	3.85	2.82^b^
SEM	0.822	6.333	0.028	5.729	1.511	4.472
*P* value	0.517	0.017	0.993	0.022	0.284	0.040
**MALONDIALDEHYDE, NMOL/MG OF PROTEIN**						
Control	25.00	48.50^a^	12.50	20.40^b^	12.00	18.76^a^
1% OMW	20.00	30.25^b^	10.85	10.00^c^	10.25	10.38^b^
2% OMW	23.00	30.00^b^	12.00	10.76^c^	10.36	10.50^b^
Antibiotic	25.10	50.25^a^	12.86	25.46^a^	12.76	20.86^a^
SEM	3.259	47.719	0.708	52.490	1.416	27.695
*P* value	0.081	0.001	0.574	0.0001	0.308	0.0001

a, b, c *Means for each trait with different superscripts differ significantly at p < 0.05; OMW, mushroom waste*.

Additionally, Table 6 shows that chicken liver, breast, and thigh tissues had values of MDA that did not vary between clusters at d 0; however, control and antibiotic groups displayed significantly higher MDA values (relative to OMW-supplemented- groups at the fifth day during refrigerated storage. This finding suggests that the mushroom in the proposed diet had a dose-dependent antioxidant function. Antibiotics have various effects on the organism's CAT and GSH-Px behaviors (19). It has been reported by Sukoyan et al. (64) that the enzyme activity of glutathione related to the antioxidant system was decreased via aminoglycosides, cephalosporin and penicillin. He also reported (64) that glutathione peroxidase activity decreased after treatment with antibiotics, while there was no change in catalase activity.

The secondary aim of this research was to explore the activity of antioxidant in mushrooms when incorporated into a planned diet. Many studies have shown strong evidence of antioxidant activity of mushroom *in vitro*. Many different polyphenolic compounds were found in mushrooms, and because of their free radical accumulative ability through *in vitro* single-electron transfer (65), they have been accepted as antioxidants. Some edible mushrooms such as Bisporus and Agaricus have been noted to own noteworthy *in vitro* antioxidant activity (66, 67).

Antioxidant activity is connected with overall phenolic content (68, 69). High content of methanol extracts in mushroom demonstrated free-radical-scavenging activity (66). For the time being, *in vivo* studies on the mentioned effects, especially on animal trial subjects like broiler chicken, are absent. Their intestines can absorb soluble, low-molecular-weight polyphenolic compounds via plasma entry and organ targeting. Even though they have low levels of circulation, with reduced net absorption and half-lives consistent with relatively rapid secretion, polyphenolic intake was followed by amplified overall antioxidant activity (70, 71).

The postprandial antioxidant potential of phenolic compounds from diverse foods has been shown in several studies (70–73). Nevertheless, the results obtained are often conflicting when the status of redox biomarkers is assessed after the intake of polyphenolic substances (71–74). In this research, it is vital to note the amplified activity of the four selenoenzymes in mushroom-fed broilers relative to control birds.

The reduction in the overall activity of glutathione was found within 5 days after a refrigerated storage step. This reduction suggested that in the tissues; the continuing development of oxidative stress should be studied further. at higher levels of OMW; the antioxidant impact was distinct, which may be due to high selenium content mushrooms (58).

The surge of antioxidant enzyme activity can be linked to the effective introduction of glutathione, a synthetic enzyme. This surge can be explained by the enzyme's high level of selenium absorption or passive glutathione sparing through reduction of the oxidative load on the cells. Even though these data seem more likely due to the reduction of MDA development in the groups provided with mushroom-supplements, further tests are needed to determine the responsible mechanism. The antioxidant properties of mushrooms may be used by the cells (), thus avoiding GSH-Px GSH, which are intracellular antioxidant systems.

*Pleurotus eryngii* mushroom at the layer ration rates of 1 percent and 2 percent significantly increased serum antioxidant enzyme activity (75). A higher content of phenolic substances, various minerals, and particularly selenium in mushrooms are responsible for this increase. In chickens, selenium fortification might increase the growth rate and the production of antioxidant enzymes (76). Finally, the researcher observed that *Pleurotus eryngii* mushroom could boost the antioxidant balance in layer chickens. The presence of phenolic compounds may cause the improvement of antioxidant status of broilers provided with various medicinal mushrooms as the phenolic acid is the main naturally occurring antioxidant component.

Nowadays, the characteristics of antioxidant for various medicinal mushrooms are well understood. Few earlier researchers indicated that the antioxidant functions of the polysaccharides and oligosaccharides present in medicinal mushrooms (8). Butylated hydroxytoluene and butylated hydroxyanisole as synthetic antioxidants may have negative effect to humans when conventionally used and thus natural antioxidant products need to be discovered (77). Tang et al. (78) reported that for their anti-inflammatory activities, the ingredients in phenolic in mushrooms might have the ability to remove the LDL oxidation. Park et al. (79) identified a fibrinolytic enzyme, which was cleansed successfully and extracted from the supernatant culture of needle mushroom. The antioxidant function depends on the various mushroom sections and varieties. Zeng et al. (80) suggested that with the highest antioxidant activity *F. velutipes* Mushroom hold a higher phenolic content. Various mushrooms have been noted to contain vitamin C and selenium, which may have an antioxidant functions role (81). A research by Lin et al. (82) showed that the mushroom *Cordyceps sobolifera* (Ascomycetes) displays antioxidant characters as a functional dietary supplement and food. However, *Agaricus brasiliensis* is considered a possible auxiliary to treat patients suffering from rheumatoid arthritis because of their ability to alleviate oxidative stress (83). Muszynska et al. (84) reported that *in vitro* culture the antioxidant and anti-inflammatory properties of *A. bisporus* biomass were extracted. In their tests, Caco-2 cell incubation with *A. bisporus* extracts resulted in declined prostaglandin F2α receptor expression and cyclooxygenase-2 compared to lipopolysaccharide (LPS) or TNF-α-activated cells. Brugnari et al. (85) also noted antioxidant activity in the *Pleurotus ostreatoroseus* (Agaricomycetes) mushroom. Kumar et al. (86) found that mushroom powder (1.2%) and 0.3% probiotics (Saccharomyces cerevisiae) in broiler diet improved the meat quality.

Data from the relevant literature showed inconsistent outcomes on the impact of natural dietary antioxidants and their antioxidant enzyme activity. (8, 56–88) Presented another study design in which human volunteers consumed grape skin extract abundant in polyphenolics, which led to amplified activity of GR and GSH-Px in erythrocytes. In another study design (34, 89, 90), discovered that with dietary supplementation of thymol and thyme oil, high level of antioxidant terpenoids, they could prevent age-induced decreased GSHPx function in the rat brain. In contrast, after natural flavonoids were given to rats, Dubost et al. (91) and Leesonet al. (92) detected a decline in the activity of GR and GSH-Px. Other cellular systems may have been influenced by mushroom compounds, indicating that further investigation of antioxidant parameters is needed in more detail.

## Conclusion

Collectively this research suggests that feeding oyster mushroom waste at a 1% level could have positive impacts on improving the performance, the humoral immune response to disease vaccines, and delayed poultry meat lipid oxidative rancidity and being more effective than 2% inclusion or antibiotic-supplemented-groups. Besides, the use of oyster mushroom waste in animal nutrition could decrease the environmental pollution. More studies are required to recognize the optimum inclusion level of oyster mushroom waste on performance enhancement and to detect the inner mechanisms of metabolic rate of lipid with oyster mushroom waste in broilers. Alternative feed resources and locally produced feeds may be useful tool for animal nutrition after COVID-19 outbreak due to expected decrease in feedstuffs for animal nutrition and increase the severity of competition between animal and human with absolute primacy given to fulfill human requirements.

## Data Availability Statement

All datasets generated for this study are included in the article/supplementary material.

## Ethics Statement

The animal study was reviewed and approved by Animal Production Research Institute. Written informed consent was obtained from the owners for the participation of their animals in this study. King Abdulaziz University, Jeddah, Saudi Arabia, approved the experimental procedures, which protect animal welfare, rights, and stress levels, and avoided any suffering or harm to creatures according to Royal Decree number M59 in 14/9/1431H.

## Author Contributions

KA set up the field work, design the experiments, and first draft of the manuscript. RH, MS, and MA contributed to the experimental setup and analyzing statistics, interpretation of results, and proofreading of the manuscript. All authors approved the final version.

## Conflict of Interest

The authors declare that the research was conducted in the absence of any commercial or financial relationships that could be construed as a potential conflict of interest.

## Abbreviations

BWG: body weight gain
CP: Crude protein
FCR: feed conversion rate
OMW: oyster mushroom waste
NDV: Newcastle disease vaccine
ME: metabolizable energy
NFE: Nitrogen free extract (starch + sugar)
KAU: King Abdulaziz University
SAS: statistical analyses software
SEM: standard error or mean
CF: crude fibre
MDA: malondialdehyde.
